# Hedonic Eating and the “Delicious Circle”: From Lipid-Derived Mediators to Brain Dopamine and Back

**DOI:** 10.3389/fnins.2018.00271

**Published:** 2018-04-24

**Authors:** Roberto Coccurello, Mauro Maccarrone

**Affiliations:** ^1^Department of Biomedical Sciences, Institute of Cell Biology and Neurobiology, National Research Council, Rome, Italy; ^2^Laboratory of Neurochemistry of Lipids, European Center for Brain Research (CERC), IRRCS Santa Lucia Foundation, Rome, Italy; ^3^Department of Medicine, Campus Bio-Medico University of Rome, Rome, Italy

**Keywords:** hedonic food, dopamine, endocannabinoids, leptin, ghrelin, orexin, insulin, lateral hypothalamus

## Abstract

Palatable food can be seductive and hedonic eating can become irresistible beyond hunger and negative consequences. This is witnessed by the subtle equilibrium between eating to provide energy intake for homeostatic functions, and reward-induced overeating. In recent years, considerable efforts have been devoted to study neural circuits, and to identify potential factors responsible for the derangement of homeostatic eating toward hedonic eating and addiction-like feeding behavior. Here, we examined recent literature on “old” and “new” players accountable for reward-induced overeating and possible liability to eating addiction. Thus, the role of midbrain dopamine is positioned at the intersection between selected hormonal signals involved in food reward information processing (namely, leptin, ghrelin, and insulin), and lipid-derived neural mediators such as endocannabinoids. The impact of high fat palatable food and dietary lipids on endocannabinoid formation is reviewed in its pathogenetic potential for the derangement of feeding homeostasis. Next, endocannabinoid signaling that regulates synaptic plasticity is discussed as a key mechanism acting both at hypothalamic and mesolimbic circuits, and affecting both dopamine function and interplay between leptin and ghrelin signaling. Outside the canonical hypothalamic feeding circuits involved in energy homeostasis and the notion of “feeding center,” we focused on lateral hypothalamus as neural substrate able to confront food-associated homeostatic information with food salience, motivation to eat, reward-seeking, and development of compulsive eating. Thus, the lateral hypothalamus-ventral tegmental area-nucleus accumbens neural circuitry is reexamined in order to interrogate the functional interplay between ghrelin, dopamine, orexin, and endocannabinoid signaling. We suggested a pivotal role for endocannabinoids in food reward processing within the lateral hypothalamus, and for orexin neurons to integrate endocrine signals with food reinforcement and hedonic eating. In addition, the role played by different stressors in the reinstatement of preference for palatable food and food-seeking behavior is also considered in the light of endocannabinoid production, activation of orexin receptors and disinhibition of dopamine neurons. Finally, type-1 cannabinoid receptor-dependent inhibition of GABA-ergic release and relapse to reward-associated stimuli is linked to ghrelin and orexin signaling in the lateral hypothalamus-ventral tegmental area-nucleus accumbens network to highlight its pathological potential for food addiction-like behavior.

## Framing fatty acids and adipocyte-derived leptin signaling within the brain reward system

The survival of all cells depends on fatty acids (FAs) that deliver energy supply and provide maintenance of the integrity of structural membranes. Particularly in the brain, FAs are present in enormous quantity, playing a key role as signaling molecules involved in neural development and defense against neuroinflammation and neurodegenerative disorders (Farooqui et al., 2007; Layé et al., 2015; Chianese et al., 2017). Acting as signaling molecules and sensors of whole-body energy status, FAs can modify the hypothalamic control of energy homeostasis (e.g., nutrient storage and mobilization) (Moullé et al., 2014). Functioning as brain region for the regulation of energy homeostasis, the hypothalamus integrates different hormonal and neuronal signals controlling appetite and body weight. Within this context, leptin provides the adipocyte-derived hormonal signaling allowing the bidirectional communication between adipose tissue and hypothalamic regulation of food intake and energy expenditure (Zhou and Rui, 2013).

Increase of energy accumulation elicits leptin release and simultaneous inhibition of orexigenic neurons expressing neuropeptide Y (NPY) and agouti-related peptide (AgRP), as well as activation of the anorexigenic proopiomelanocortin (POMC) neurons within the hypothalamic arcuate nucleus (ARC). Thus, leptin release produces satiety effects and oxidation of fat depots that prevents further synthesis and release of leptin (Coll et al., 2007). The bidirectional communication throughout the brain-adipose axis allows a homeostatic balance between energy intake and expenditure, which starts to be deranged by excessive accumulation of white adipose tissue. However, despite very high levels of circulating leptin obese individuals cannot rely on leptin signaling neither to reduce appetite nor to increase energy expenditure. This condition is well-known as “leptin resistance” and develops gradually as function of body adiposity, from residual sensitivity to exogenous leptin to almost total suppression of brain leptin sensitivity (Lin et al., 2000).

The excessive eating of dietary fat not only dysregulates the homeostatic control of feeding behavior and body weight, but has also a great importance in the derangement of the brain hedonic system. Overeating is a maladaptive behavior that is triggered and sustained by the escalation of easily accessible palatable or hyperpalatable (e.g., high fat, sugar-rich, and often salty diet) food, that exacerbates energy intake and vulnerability to hedonic experience.

Leptin function and signaling link the regulation of energy homeostasis to the incentive and reward value of food and nutrients. Indeed, leptin-mediated effects are not limited to feeding circuits but extend over involving hedonic, cognitive and stress neuronal circuits (Morrison, 2009; Farr et al., 2015). The key point to understand the two faces of energy homeostasis (i.e., energy loss or satiation and energy intake or hunger) is to look at the intricate puzzle where nutritional status and reward value of food coexist.

It is recognized that starvation or food restriction significantly enhances motivation for rewarding stimuli, including craving for palatable food and drugs of abuse (Carr, 2007). Leptin can exert a dual action by reducing food intake and also motivation to attain rewards (Figlewicz et al., 2001, 2004, 2006; Carr, 2007; Shen et al., 2016). Reinforcing properties of both palatable food (Hommel et al., 2006) and addictive substances (Shen et al., 2016) are encoded by dopamine (DA) transmission within the mesocorticolimbic network, encompassing the projection neurons of the ventral tegmental area (VTA) in the midbrain that relays DA-ergic signals to the ventral striatum (nucleus accumbens, NAc), amygdala and prefrontal cortex (PFC). Although DA shows to be a neural communication system shared by food and drug seeking, a perfect isomorphism between these two processes would be an oversimplification.

Here, we will assume that drugs and palatable foods are potent reinforcers that disrupt the brain mechanisms underlying synaptic plasticity and energy homeostasis, that show common vulnerabilities and pathophysiological aspects (Volkow et al., 2011b).

Thus, the highly conserved mesocorticolimbic DA circuit plays a fundamental role in the assignment of motivational/rewarding value to biologically relevant stimuli (Kelley and Berridge, 2002). VTA, NAc, amygdala, PFC, and the lateral hypothalamus (LH) are all brain regions activated by palatable food. Drugs of abuse and palatable food share the ability to drastically shape the mesocorticolimbic DA circuit, altering synaptic strength and increasing DA tone, and in particular NAc DA transmission (Kenny, 2011). Undisturbed access to palatable food overtakes satiety signals, prolongs meal duration and generates neuroadaptations whereby both hedonic eating and addictive drugs shift effective doses or body weight to a higher set point. However, a major affinity between repeated drug abuse and habitual consumption of energy-rich palatable food is the compulsive nature of the underlying activities. Drug addicts lose control over their actions showing impaired inhibition of seeking behavior and consummatory processes. Similarly, repeated exposure to palatable food can lead to compulsive eating that is characterized by inadequate control over food provisions and conditioning to food-associated stimuli, in spite of the negative consequences (Teegarden and Bale, 2007; Hoebel et al., 2009; Dalley et al., 2011). In the analysis of palatable food addiction-like eating behavior the contribution of the surrounding environment should never be undervalued. The current situation of Western societies (an extremely obesogenic scenario) is characterized by sedentary activities, large food provisions and effortless access to energy-rich food.

The communication between NAc and LH has great relevance for energy homeostasis and, consequently, for sensing the nutritional status and for encoding the reward value of food. Being at the crossroad between the mesolimbic reward circuit and ARC homeostatic system, LH contributes to homeostatic feeding and incentive salience of food.

**Figure 1:**
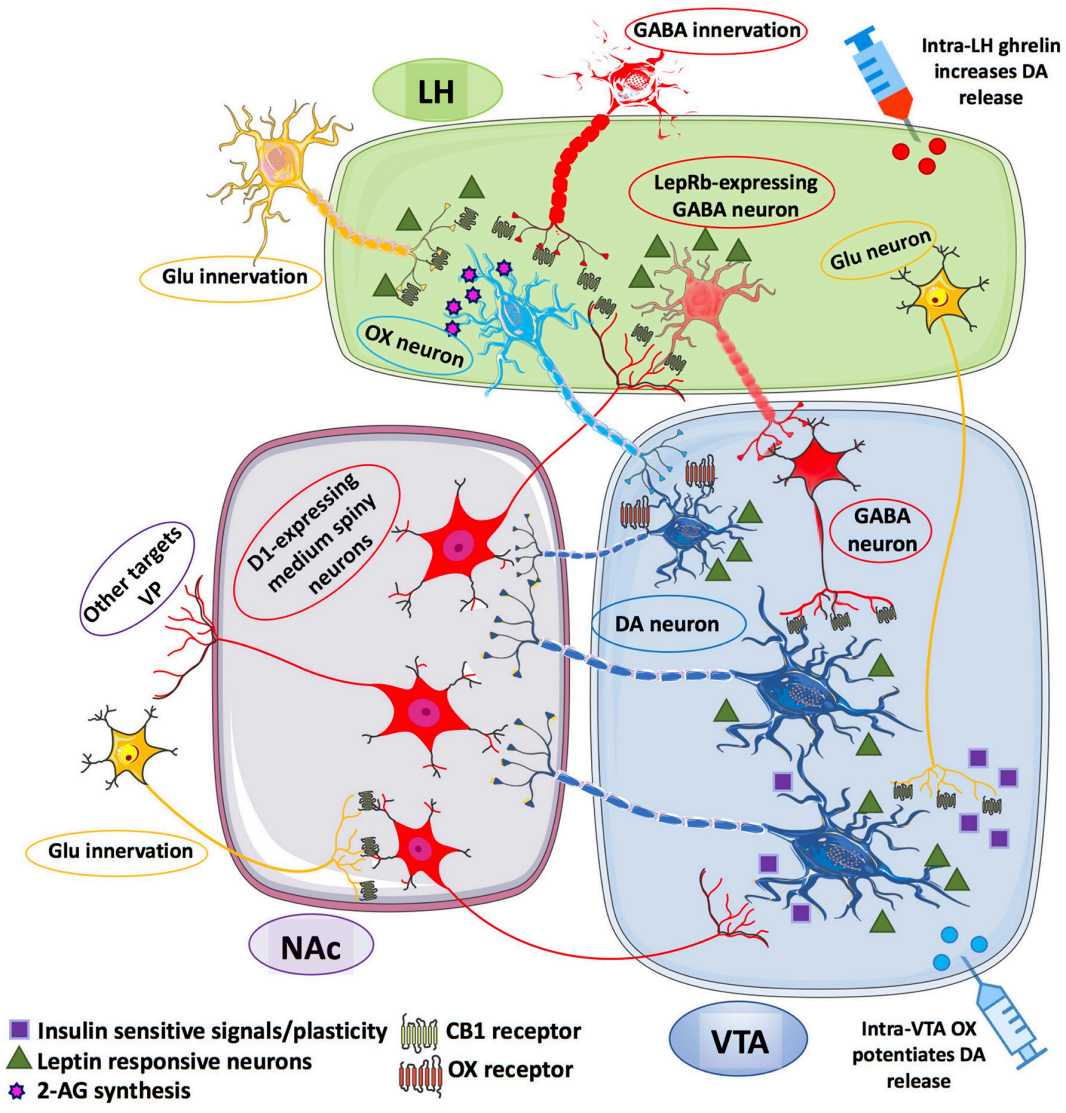
Schematic representation of the information flow among the afferent and efferent neural pathways connecting lateral hypothalamus (LH), ventral tegmental area (VTA) and nucleus accumbens (NAc). LepRb-expressing GABA neurons within the LH receives inhibitory inputs from D1-expressing medium spiny neurons (MSNs) of the NAc. In turn, LH GABA neurons can inhibit VTA GABA-ergic neurons that disinhibit VTA DA neurons that may project onto D1-expressing MSNs of the NAc. LH OX neurons receives excitatory inputs from glutamatergic (Glu) innervation under the inhibitory control of presynaptic CB_1_ receptors, also located on presynaptic GABA-ergic terminals that synapse OX neurons within the LH. OX neurons are thought to form local microcircuits by synapting VTA DA neurons establishing contacts with D1-expressing MSNs of the NAc, which project back to LepRb-expressing GABA neurons. Glu-ergic innervation from LH to VTA DA neurons is also illustrated as relevant instance of insulin- and leptin-sensitive information processing within the LH-mesolimbic DA circuit. The Glu-ergic pathway, along with presynaptic CB_1_ receptors on Glu-ergic terminals contacting NAc MSNs illustrate an additional key component of the fine-tuned regulation of VTA DA neurons. Putative Glu-ergic neurons (yellow), GABA-ergic neurons (red), OX neurons (light blue) and DA-ergic neurons (blue).

Two orexigenic neural populations of LH participate in these processes: the neuropeptide melanin-concentrating hormone (MCH) and the orexins (OX) population, establishing a communication system with NAc and VTA, respectively (Georgescu et al., 2005; Cason et al., 2010). Leptin can suppress feeding by acting on GABA-ergic LH leptin-expressing (LepRb) neurons, and its anorectic action increases VTA tyrosine hydroxylase expression and mesolimbic DA content (Leinninger et al., 2009) (Figure 1). Moreover, leptin modulates LepRb-expressing neurons of DA-ergic VTA and its administration reduces feeding and DA release within the NAc, thus exerting a suppressive action against food-induced reward (Fulton, 2000; Krügel et al., 2003; Hommel et al., 2006). Instead, knock-down of LepRb in the VTA enhances preference toward sucrose and high fat diet (HFD) (Hommel et al., 2006). Hence, chronic consumption of palatable food is a maladaptive behavior that is sustained by an alteration of the brain reward system with disruption of DA signaling, upregulation of orexigenic signals (Huang et al., 2004; Gaysinskaya et al., 2007) or blunted sensitivity to satiety signals such as leptin and cholecystokinin (Lin et al., 2000; Savastano and Covasa, 2005).

## Craving for food pleasure: the good and the bad of dietary fat intake

As described above, the activation of DA signaling within the mesocorticolimbic reward circuitry represents a common neural code linking one another drug abuse and hedonic eating. Nevertheless, palatable food is craved because of its high fat content and composition.

Hence, it becomes critical to understand the relationship between (types of dietary) fats, satiety and hedonic overeating. It is recognized that ingestion of saturated fatty acids (SFAs) leads to higher adipose tissue accumulation, as well as to insulin resistance and activation of inflammatory pathways like macrophage infiltration (Enos et al., 2013), whereas the ingestion of monounsaturated fatty acids (MUFAs) and polyunsaturated fatty acids (PUFAs) allows a tight control on body weight fluctuations via mechanisms involving thermogenesis, expression of satiety-associated neuropeptides and fat oxidation (Dziedzic et al., 2007; Krishnan and Cooper, 2013). The satiating power of different forms of FAs over the hypothalamic homeostatic system has been elegantly demonstrated when oleic acid (OA) was directly infused (i.e., intracerebroventricularly) in the central nervous system (CNS), curbing food intake via the decreased expression of NPY (Obici et al., 2002) or inhibition of carnitine palmitoyltransferase-1 (CPT1)-dependent fat oxidation (Obici et al., 2003; Coccurello et al., 2010).

Dietary PUFAs can directly affect and remodel fatty acid composition of plasma membranes, and ingestion of *n*-3 PUFA (eicosapentaenoic acid [EPA] and docosahexaenoic acid [DHA])-enriched diet can help to rebalance the *n*-6 to *n*-3 ratio. The latter is sharply exacerbated by the current Western diet characterized by the massive increase in dietary *n*-6 PUFAs. Of note, it is recognized that an unbalanced *n*-6 to *n*-3 ratio underlies development of brain and systemic inflammation, obesity, cancer and cardiovascular disease (Simopoulos, 2006; Grant et al., 2011).

Within this context, a special class of lipids might be accountable not only for dietary fat-induced overweight but also for hedonic eating and addiction-like eating behavior: endocannabinoids (eCBs). These are PUFA-derived lipid mediators, produced from cell membrane phospholipid precursors having key metabolic functions not only in energy homeostasis (positive energy intake/balance, anabolic control and body weight gain), but also in the hedonic aspects of feeding (Maccarrone et al., 2010a; D'Addario et al., 2014; Mancino et al., 2015). The so-called eCB system (ECS) is composed of: (i) the two main eCB-binding cannabinoid CB_1_ and CB2 receptors, which are both metabotropic receptors coupled to Gi/o proteins; (ii) their best characterized endogenous ligands, *N*-arachidonylethanolamine (anandamide (AEA)) and 2-arachidonoylglycerol (2-AG); and (iii) the enzymes and proteins responsible for eCB biosynthesis, transport, accumulation and degradation (Pertwee, 2006; Di Marzo, 2008; Maccarrone et al., 2010a). Both AEA and 2-AG are PUFAs derivatives, and in particular they are the *N*-ethanolamine and the glyceryl ester, respectively, of the *n-*6 PUFA arachidonic acid (ARA, 20:4) (Devane et al., 1988; Mechoulam et al., 1995). Notably, AEA can be accumulated into adiposomes (Oddi et al., 2008), and variations in dietary FAs intake are mirrored by changes in eCB levels (Berger et al., 2001). Then, dietary intake of PUFAs and appropriate balance of *n*-6 to *n*-3 ratio become essential for eCB biosynthesis, and for the impact of eCB levels and their neural control on energy metabolism and hedonic eating (Artmann et al., 2008; Wood et al., 2010).

More than dietary fat calories, the percentage of *n-*6 linoleic acid (LNA, 18:2), a precursor of ARA, in the diet appears responsible for body weight gain and increase of ARA, AEA, and 2-AG levels in liver, red blood cells and white adipose tissue (Alvheim et al., 2014). Both AEA and 2-AG production is highly susceptible to dietary consumption of LNA, and to synthesis of the eCB substrate ARA. Driving the production of AEA and 2-AG, dietary lipids with a disproportionate amount of *n*-6 PUFAs lead to inflammation, excessive energy intake and storage, overall favoring adiposity. In addition, it leads to higher susceptibility to the rewarding value of palatable food.

Oral exposure to LNA increased AEA and 2-AG levels in rat jejunum, as well as preference for high LNA-rich fat food that was abolished by CB_1_ receptor blockade (DiPatrizio et al., 2013). Although to different extents, both AEA and 2-AG levels increase during fasting in both homeostatic and hedonic brain regions, and administration of AEA within the hypothalamus (Jamshidi and Taylor, 2001), or of 2-AG within the NAc (Soria-Gómez et al., 2007), stimulates feeding, palatable food intake and appetite motivation through the activation of CB_1_ receptors (Jamshidi and Taylor, 2001; Kirkham et al., 2002; Shinohara et al., 2009). Notably, activation of CB_1_ receptor underlid “liking” and increased intake of a sucrose rewarding solution after direct AEA infusion into the NAc shell (Mahler et al., 2007).

Thus, ECS activation favors anabolism over catabolism, and its energy preservation function appears vital under conditions of low food provisions or of uncertainty about food availability. Conversely, it becomes detrimental with large, virtually unlimited, availability of energy dense palatable food. An increase of 2-AG plasma levels was observed in obese patients after the ingestion of palatable hedonic food, whereas 2-AG plasma levels were decreased after consumption of non-palatable food (Monteleone et al., 2016). Hence, eCBs and particularly 2-AG signaling appears enhanced in response to reward-associated palatable food in obese subjects (Monteleone et al., 2016).

## Endocannabinoids and related bioactive lipid mediators across homeostatic and hedonic eating

eCBs are retrograde messengers able to cross the synaptic cleft and inhibit neurotransmitter release at excitatory or inhibitory synapses. The main evidence for an interconnected network that engages eCB signaling and DA-ergic transmission able to influence the rewarding aspects of eating is provided by the dense distribution of CB_1_ receptors within the mesolimbic and mesocortical areas, such as NAc, PFC and amygdala (Di Marzo et al., 1998). In particular, they are expressed in glutamatergic and GABA-ergic presynaptic terminals impinging on DA neurons that are able to synthetize and release eCBs (Mátyás et al., 2008). Indeed, despite the lower concentration of CB_1_ receptors in VTA than in striatum (i.e., the major mesolimbic projecting area), a CB_1_-dependent mechanism is implicated in the firing rate of VTA DA-ergic neurons (Maldonado et al., 2006). Specifically, activation of CB_1_ receptors on glutamatergic terminals disinhibit the activity of DA neurons within VTA by inhibiting GABA-ergic neurons that send inputs from NAc to VTA (Maldonado et al., 2006; Parsons and Hurd, 2015). Given the well-known role of eCB transmission in shaping synaptic plasticity via short- and long-term depression (STD and LTD, respectively) and depolarization-induced suppression of excitatory (DSE) or inhibitory (DSI) transmission, eCB signaling can finely adjust the weight of the different incoming inputs, and then calibrate the firing of DA neurons. The increase of AEA and 2-AG tone enhances also DA-ergic transmission at NAc level and this facilitatory effect is mediated by CB_1_ receptors (Solinas et al., 2006; De Luca et al., 2014). Hedonic responses elicited by sucrose are facilitated by cannabinoid-dependent increase of DA release in the NAc shell, which is reduced by CB_1_ receptor blockade (Melis et al., 2007). Thus, palatable rewarding food elicits DA release via a CB_1_ receptor-mediated effect (Melis et al., 2007; De Luca et al., 2012). Several studies have underlined the role of CB_1_ receptor-dependent LTD in the derangement of neural plasticity in brain areas involved in processing of drug-associated reward (Sidhpura and Parsons, 2011). Repeated exposure to cocaine decreases the GABA-ergic inhibition in VTA DA neurons via a CB_1_-mediated LTD mechanism, which may account for sensitization to cocaine rewarding effects and cocaine-seeking behavior (Pan et al., 2008). A mechanism leading to facilitation of DA release in the NAc was demonstrated by the cocaine-induced secretion of 2-AG and activation of presynaptic CB_1_ receptors on GABA terminals, followed by decrease of GABA release and disinhibition of VTA DA neurons (Wang et al., 2015). On this basis, future studies are deemed necessary to determine the role of eCB signaling in food-mediated long-term plasticity at inhibitory and excitatory synapses within the mesolimbic circuit and VTA (Figure 1).

As discussed above, leptin is pivotal for the brain reward system, and understanding leptin signaling is needed to decipher food addiction-like behavior. The reciprocal regulation between eCB and leptin signaling in the hypothalamus, where defective leptin is associated with higher eCB levels (Di Marzo et al., 2001), prompted to interrogate the possibility that leptin and eCBs may regulate each other in a reciprocal fashion at LepRb-expressing neurons of DA-ergic VTA neurons. However, while there is sparce evidence for a similar reciprocal interaction in mesolimbic DA neurons, new data on a negative regulation of leptin anorexigenic effect via the stimulation of CB_1_-mediated signaling within the hypothalamic ARC has been provided (Palomba et al., 2015). Leptin can exert trophic actions, as shown by its ability to affect neural plasticity and remodel synaptic connectivity in leptin-deficient mice (Pinto, 2004). An increase of CB_1_ receptor-expressing GABA-ergic inhibitory input to LH, along with an increase of 2-AG biosynthesis, has been observed in leptin-deficient (*ob*/*ob*) mice, both events leading to a drastic eCB-mediated disinhibition of LH OX neurons (Cristino et al., 2013). The enhanced retrograde inhibition of inhibitory inputs was mainly mediated by 2-AG (Becker et al., 2017), through a mechanism that boosts OX signaling; instead, leptin administration can reinstate the full control of energy homeostasis by LH (Cristino et al., 2013).

Decoding the complex relationship between leptin, OX and eCB within the mesolimbic network might open new avenues for the understanding of food-addiction behaviors and eating disorders. In this context, it should be mentioned that increased plasma levels of AEA have been found in women with anorexia nervosa and binge-eating disorders, where increased sensitivity to the rewarding aspects of palatable food dependent by eCB tone is hypothesized (Monteleone et al., 2005). Notably, binge-like eating behavior is blocked by CB_1_ receptor inverse agonism/antagonism in female rats, an effect that may be associated with the reduction of DA release within the NAc shell (Scherma et al., 2013, 2014) (Figure 1).

### Dietary fats: control and derangement of homeostatic eating

Consumption and composition of dietary fats appear critical factors to explain the derangement of homeostatic eating and overpowering by hedonic brain circuits. Decreased DA activity within the striatum, NAc and VTA and consequent deficit in reward processing is associated with compulsive eating and enhanced sensitivity to the hedonic properties of palatable food (Wang W.G. et al., 2001; Cordeira et al., 2010; Johnson and Kenny, 2010). Protracted intake of palmitic acid-rich diet blunts mesolimbic DA function, which instead is not affected by the ingestion of olive oil (Hryhorczuk et al., 2016). *N*-oleoylethanolamine (OEA or NAE 18:1), *N*-palmitoylethanolamine (PEA or NAE 16:0), *N*-linoleoylethanolamine (LEA or NAE 18:2), *N*-stearoylethanolamine (SEA or NAE 18:0), *N*-eicosapentaenoylethanolamine (EPEA) and docosahexaenoylethanolamide (DHEA) are structural analogs of AEA (Schmid and Berdyshev, 2002; Hansen, 2010; Pertwee, 2015). For all these NAEs the biosynthetic pathways initiate from the common precursor *N*-acylphosphatidylethanolamine (NAPE). However, while AEA (NAE 20:4) derives from the precursor ARA, other NAEs such as PEA and OEA derive from the saturated fatty acid palmitic acid and from the *n*-9 monounsaturated oleic acid (OA), respectively (Hansen, 2010; Ueda et al., 2010). OEA is a well-characterized satiety factor and anorexiant agent, whose endogenous levels are directly modulated by the ingestion of dietary fatty acids in the small intestine (luminal layer) via the generation of OA-containing NAPE precursor (Rodríguez de Fonseca et al., 2001; Hansen, 2010; Ueda et al., 2010). OEA belongs to a special kind of satiety factors, whose action depends on the nutritional state. Indeed, OEA decreases meal frequency and meal size in case of food deprivation, and induces early satiety in non-food deprived animals (Coccurello et al., 2008; Gaetani et al., 2010; Provensi et al., 2014; Romano et al., 2014). Beyond its role as satiety signal and the brain mechanisms and receptors translating its action (Provensi et al., 2014), there is growing evidence that OEA can be implicated in fat sensing and modulation of hedonic food intake in reward circuitry (Piomelli, 2013; Tellez et al., 2013). Not only uncontrolled, but even short-term intake of dietary fat (60 kcal%) disrupts the enzymatic pathways involved in OEA synthesis, and consequently abolishes intestinal OEA mobilization (Igarashi et al., 2015). Moreover, OEA administration inhibits nicotine-evoked activation of DA neurons (Melis et al., 2008) and nicotine- and ethanol-associated reward (Mascia et al., 2011; Bilbao et al., 2016). Along with the evidence that OEA engages brain histamine signaling to induce satiety (Provensi et al., 2014), there are data supporting the idea that this lipid mediator can reverse HFD-induced decrease of striatal DA signaling (Tellez et al., 2013). The effect of OEA administration on drug-associated addictive behaviors further corroborates the interplay between lipid mediators, food addiction, DA transmission and drug-seeking behavior. From this perspective, OEA may be viewed as a lipid messenger able to rebalance DA-dependent hypersensitivity to food reward in high fat-fed animals. Accordingly, instead of reducing intake OEA administration was shown to increase the intake of less palatable low-fat emulsions in HFD-fed animals via a DA-mediated mechanism (Tellez et al., 2013). This is the best available demonstration that a lipid-derived satiety factor involved in homeostatic feeding (Provensi et al., 2014) can “cross the border” and impinge on mechanisms associated with hedonic processing (e.g., DA signaling), thus modulating the assignment of reward value to food. Notably, OEA plasma levels are differently associated with food-related cortical activation depending on the assessment in healthy or obese individuals (Grosshans et al., 2014). Indeed, while higher OEA plasma levels were found to positively correlate with higher brain activity of the insula region, an inverse relationship between them was found in obese subjects (Grosshans et al., 2014). Direct brain infusion of monounsaturated OA has been shown to curb food intake (Obici et al., 2002; Coccurello et al., 2010), while prolonged intake of saturated fat (i.e., palmitic acid) reduced DA signaling and sensitivity to the rewarding effect of amphetamine administration (Hryhorczuk et al., 2016). On the other hand, direct infusion of OA within the mesolimbic circuit (i.e., VTA) reduced both reward-induced motivation to obtain high palatable high-fat/high-sucrose food, and firing of DA-ergic neurons (Hryhorczuk et al., 2017). The latter study provides evidence that DA neurons can bind and transport FAs, supporting the concept that the same neurons can act as lipid sensors (Hryhorczuk et al., 2017) and show vulnerability to long-term exposure to dietary fats and their composition. The main implication of studies discussed in sections Craving for Food Pleasure: The Good and the Bad of Dietary Fat Intake and Endocannabinoids and Related Bioactive Lipid Mediators Across Homeostatic and Hedonic Eating are schematized in Tables 1, 2.

**Table 1:** Selected studies that investigated the interplay between DA and eCB signaling.

**DA and eCB signaling interplay**	**VTA DA activity**	**NAc DA activity**	**Liability to hedonic food**	**Study**
CB_1_-mediated activation Glu-ergic terminals reduces excitatory inputs on GABA-ergic neurons to VTA	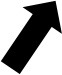		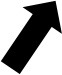	Maldonado et al., 2006; Parsons and Hurd, 2015
Increase of AEA and 2-AG tone/CB_1_-dependent facilitatory effect		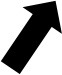	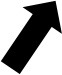	Solinas et al., 2006; De Luca et al., 2014
eCB-dependent DA release		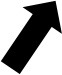	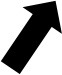	Melis et al., 2007
CB_1_-dependent hedonic food responses/palatability		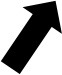	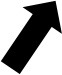	Melis et al., 2007; De Luca et al., 2012;
Reduced GABA-ergic inhibition of VTA DA neurons after repeated cocaine exposure	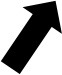		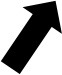	Pan et al., 2008
Cocaine-induced 2-AG secretion and decrease of GABA-mediated inhibition	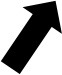		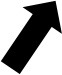	Wang et al., 2015

*Main effects on VTA and NAc DA activity and liability to hedonic food consumption are schematized. Ascending arrows denote increase*.

**Table 2:** Selected studies showing the crosstalk between eCB signaling, leptin and ghrelin.

**eCB signaling/ Leptin/Ghrelin**	**Liability to hedonic food**	**Study**
Increase of CB_1_ receptor-expressing GABA-ergic neurons and 2-AG synthesis in leptin deficient (*ob*/*ob*) mice	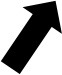	Cristino et al., 2013
Increased AEA plasma levels	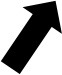	Monteleone et al., 2005
CB_1_ receptor inverse agonism abolishes binge-like eating	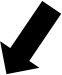	Scherma et al., 2013, 2014
Palmitic acid-rich diet blunts DA mesolimbic function	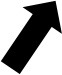	Hryhorczuk et al., 2016
OEA abolishes activation of DA neurons	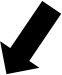	Melis et al., 2008
OEA increases intake of low palatable fat emulsion in HFD-fed mice	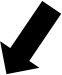	Tellez et al., 2013
Inverse correlation between insula activation and OEA plasma levels in obesity	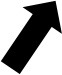	Grosshans et al., 2014
Direct OA brain infusion	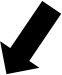	Obici et al., 2002; Coccurello et al., 2010
Direct OA VTA infusion decreases DA signaling and reward-seeking behavior	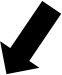	Hryhorczuk et al., 2017
Brief exposure to HFD blunts the anorectic effects induced by OA administration	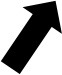	Morgan et al., 2004
Higher ghrelin plasma levels in anorexia nervosa lean patients/ghrelin resistance	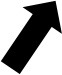	Tanaka et al., 2003; Miljic et al., 2006
Intra-VTA chronic ghrelin infusion reinstates cue-induced responses for palatable food	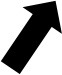	St-Onge et al., 2016
Intra-VTA, intra-NAc or intra-LH ghrelin infusion	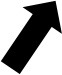	Naleid et al., 2005; Szentirmai et al., 2007; Skibicka et al., 2011; Kanoski et al., 2013
Intra-LH ghrelin induces DA release; intra-VTA OX increases food-induced DA release	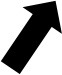	Cone et al., 2014
Ghrelin-induced neural activity linked to food expectation in satiated subjects	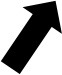	Overduin et al., 2012; Simon et al., 2017

*Main effects on liability to hedonic food consumption are schematized. Ascending and descending arrows denote increase and decrease, respectively*.

## The ghrelin-brain axis and endocannabinoid signaling: reshaping the hedonic food network

Depending on fat composition, the ingestion of dietary fat can elicit either satiety or hyperphagia, and one of the most intriguing issues about the contribution of dietary fat in eliciting opposite effects is the crosstalk between peptides that homeostatically regulate feeding and DA-driven food reward.

Ghrelin is a 28 amino acids peptide hormone secreted in the upper gastrointestinal tract (stomach and duodenum) by specialized endocrine cells within the gastric oxyntic (fundus) mucosa. These endocrine cells take different names: X/A-like type cells in rodents, and P/D1-type cells in humans (Date et al., 2000; Mizutani et al., 2009). Ghrelin is the ligand for the growth hormone secretagogue receptor (GHSR)1a, a G-protein coupled receptor that stimulates growth hormone (GH) release from the anterior pituitary gland (Howard et al., 1996). Beyond the pituitary gland and gastrointestinal tract, the GHSR1a is expressed in several peripheral tissues such as pancreas, adrenal gland, adipose tissue, thyroid, myocardium and immune cells and, within the brain, in hypothalamic ARC, LH, ventromedial (VM), and paraventricular (PV) nuclei, as well as in hippocampus, substantia nigra pars compacta (SNpc), VTA, raphe nuclei, and NTS (Guan et al., 1997; Gnanapavan et al., 2002; Hou et al., 2006).

The powerful orexigenic action of ghrelin is a fundamental homeostatic function that is fulfilled by the unique capacity of this hormone to pass through the brain blood barrier and gain access to the brain, where it signals the (negative) fuel status of the organism and modifies energy intake, nutrient partitioning and expenditure accordingly (Tschöp et al., 2000; Nakazato et al., 2001). Ghrelin accomplishes its goal by increasing the expression of orexigenic *NPY* and *AgRP* neurons in the ARC, and by amplifying the frequency of GABA release onto anorexigenic proopiomelanocortin (*Pomc*) neurons with consequent decrease of their firing activity (Cowley et al., 2003). Moreover, ghrelin secretion is fine-tuned according to circadian eating time with higher plasma levels conveying hunger signals and anticipating each mealtime. Yet, ghrelin action is not limited to hunger stimulation, increase of food intake and maintenance of body weight. Ghrelin exerts indeed a tight control on glucose homeostasis (e.g., hyperglycemia) and adipogenesis and inhibits insulin secretion (Broglio et al., 2001).

Dysregulation of ghrelin signaling is reported in eating disorders, with higher ghrelin plasma observed in lean patients suffering from anorexia nervosa (AN) and in patients with the binge-eating variant of AN (Shiiya et al., 2002; Tanaka et al., 2003). The co-occurrence of food intake restriction and increase of ghrelin signaling has suggested the notion that AN may be a condition associated with “ghrelin resistance” (Miljic et al., 2006). The involvement of ghrelin in eating disorders calls for our attention toward the role of ghrelin in addictive behaviors and derangements of reward mechanisms. As noted, the large expression of GHSR1a receptors in the VTA and mesolimbic circuit (Guan et al., 1997) is a solid clue to support the idea that ghrelin signaling can encode food-associated reward. Accordingly, ghrelin infusion within the VTA, NAc or LH significantly increases feeding of palatable food (Naleid et al., 2005; Szentirmai et al., 2007) as well as incentive motivation to eat when microinjected in the same VTA or in ventral hippocampus (Skibicka et al., 2011; Kanoski et al., 2013). Of note, conditioned place preference for high rewarding/palatable food (i.e., chocolate) can be induced by ghrelin administration and abolished in calorie-restricted animals by blocking the GHSR1a receptors (Perello et al., 2010). The visual presentation of food images to humans administered with ghrelin selectively activates a motivational brain circuit involved in the appetitive component or incentive value of food, including the orbitofrontal cortex, amygdala and striatum (Malik et al., 2008). In a functional magnetic resonance imaging task carried out to assess human responses to food pictures, ghrelin administration mimicked the effects produced by 16-h fasting as regards the activation of corticolimbic system (Goldstone et al., 2014). The consumption of high palatable food in satiated healthy volunteers increases ghrelin plasma levels and concomitant decrease of cholecystokinin secretion (Monteleone et al., 2013), which elegantly demonstrates that ghrelin signaling associated with hedonic eating is activated in reciprocal fashion with respect to satiety signaling. However, ghrelin signaling has not been definitely linked to hedonic eating until the demonstration that intracerebroventricular or VTA ghrelin infusion was able to elicit DA release in the NAc (Abizaid et al., 2006; Jerlhag et al., 2007). The neural substrates (i.e., mesolimbic circuit) involved in the action of ghrelin overlap partially with those implicated in drug addiction and food consumption. Moreover, the ability of ghrelin to increase reward-seeking and motivation to eat appears to be mediated by the VTA (King et al., 2011; Skibicka et al., 2011), and blockade of ghrelin signaling reduces the rate of operant behavior to obtain sucrose reward to the same level of satiated animals (Skibicka et al., 2012). The phasic activation of DA VTA neurons during a food-seeking operant task motivates actions directed toward positive reinforcement and amplifies the value of reward-associated cues (Adamantidis et al., 2011). From this view, ghrelin and DA might concur to play the role of “teaching” signals that are activated when a non-expected reward is delivered but also when cues predicting food delivery are presented, thus facilitating the switching from the reinforcer (e.g., food) to environmental cues in the form of reward predictors (Volkow et al., 2011a; Schultz, 2016).

Within this context, the LH-VTA-NAc neuroanatomical network can help to disclose the functional relationship between not only ghrelin, DA and OX, but also eCB signaling. Notably, OX neurons in LH are specifically activated by environmental cues associated with rewarding stimuli, both synthetic (drugs) or natural (Harris et al., 2005). There at least two functionally relevant LH-VTA local circuits in which DA, ghrelin and OX transmission regulate food reward processing. First, the OX-induced stimulatory effect on glutamatergic projection to VTA DA neurons links together ghrelin-activated OX neurons and increased activity of VTA DA neurons, a circuit potentiated by highly salient reinforcers such as rewarding food (Borgland et al., 2009). Secondly, the activity of VTA DA neurons can also be indirectly stimulated by the strong GABA-ergic inhibitory output of LH projection onto VTA GABA-ergic neurons. Indeed, by the interposition of VTA GABA-ergic neurons, the inhibitory output from LH disinhibits DA release from VTA DA cells to the NAc and mediates positive reinforcement and reward-associated appetitive behaviors (Jennings et al., 2015; Barbano et al., 2016; Nieh et al., 2016). Next, the stimulation by VTA DA neurons of NAc GABA-ergic D1 receptor-expressing medium spiny neurons induces feedback inhibition of LH GABA-ergic neurons. Consequently, the negative feedback loop is activated and the motivational drive is reduced or even ceased. Intra-LH, but not VTA, ghrelin administration stimulates DA release and intra-VTA OX-A administration potentiates the magnitude of phasic DA evoked by food retrieval (Cone et al., 2014). Thus, the LH-VTA local circuit and OX neurons play a key role in the potentiating effects of ghrelin on food-evoked DA release. However, the reciprocal interaction between DA and ghrelin signaling can be even more complex. Indeed, the recent discovery (Kern et al., 2012) that DA D2 receptors (D2Rs) and GHSR1a can form functional heteromers has shed a new light onto the mechanistic action of GHSR1a-mediated signaling on DA-dependent regulation of feeding. In the latter study, a subset of hypothalamic neurons was found to co-express D2Rs and GHSR1a, and to generate heteromer-specific receptor-receptor physical interaction with distinct functional effects. GHSR1a:D2Rs heteromers change the classical D2Rs-mediated signaling, and pharmacological blockade of GHSR1a suppresses the anorexigenic effects induced by D2Rs stimulation (Kern et al., 2012). The demonstration that D2Rs-induced anorexia depends upon GHSR1a:D2Rs interaction, and in particular by the allosteric modulation by GHSR1a upon D2R signaling provides an important insight into the mechanisms responsible of reward-seeking behavior and hedonic eating. The potential existence of GHSR1a:D2Rs functional heteromers also in the LH-VTA-NAc circuit might help to explain the reduced or even abolished craving for palatable food and drug of abuse, as well as inhibition of NAc DA release induced by systemic or intra-VTA pharmacological blockade of GHSR1a (Abizaid et al., 2006; Jerlhag et al., 2010, 2011). On this basis it has been suggested that chronically elevated ghrelin levels as in Prader-Willi syndrome may reduce the expression of GHSR1a on plasma membrane and concentration of GHSR1a:D2Rs heteromers, leading to decrease of D2R-dependent satiety signals (Kern et al., 2012). Moving from hyperphagia to eating disorders, it is intriguing that abnormal ghrelin plasma levels reported in binge-type bulimic subjects and in lean patients with AN might alter the balance of GHSR1a:D2Rs signaling, thus producing effects upon reward-based eating that depend on the allosteric interaction between GHSR1a and D2R. Incidentally, these mechanisms offer also additional explanations for the frequently observed co-morbidities among binge-type eating disorders and drug of abuse (Gregorowski et al., 2013). OX signaling is also involved in learning stimulus-reward associations, as demonstrated by the possibility to reinstate an extinguished drug-associated place preference via the stimulation of LH OX neurons (Harris et al., 2005) (Figure 1).

Processing of environmental cues associated with palatable food is of great importance for determining excessive consumption of caloric food, and for overriding signals that convey homeostatic satiety. Mutual relationships between OX, ghrelin and eCB signaling can account for processing of the complex cluster of stimuli in the food environment (i.e., food-predictive cues). Thus, meal initiation can be elicited by exposure to a food cue in sated rats via a direct ghrelin infusion into the ventral hippocampus (Kanoski et al., 2013). Moreover, pharmacological or genetic disruption of ghrelin signaling abolishes cue-induced eating (Walker et al., 2012). As mentioned above, ghrelin increases in healthy subjects the coordinated neural response evoked by food pictures in different brain regions belonging to the hedonic eating circuitry, such as amygdala, striatum, orbitofrontal cortex and insula (Malik et al., 2008). Food expectation and meal anticipatory behavior is reduced in ghrelin receptor deficient mice (Davis et al., 2011a), and ghrelin signaling has been recently demonstrated to affect hedonic food consumption by directly enhancing in satiated healthy subjects the neural activity associated to the expectation or motivation (i.e., “wanting”) for food reward (Overduin et al., 2012; Simon et al., 2017). Moreover, ghrelin signaling was shown to coordinate phasic DA activity in the NAc evoked by presentation of a positive conditioned stimulus that predicts delivery of rewarding food (Cone et al., 2015).

However, besides DA ghrelin orexigenic action is modulated by the functional interaction with eCB-mediated signaling. Indeed, ghrelin effect is inhibited by intra-hypothalamic infusion of subthreshold doses of the CB_1_ receptor antagonist/inverse agonist SR141716A (Tucci et al., 2004). Plasma levels of 2-AG increase not only during food deprivation but also during consumption of hedonic food in satiated healthy subjects (Monteleone et al., 2012). Together with the increase of 2-AG levels, consumption of high palatable food produced also an increase of ghrelin plasma levels that did not decrease after ingestion, unlike what occurred in the same subjects after consumption of non-palatable food. Ghrelin potentiates the neural responses evoked by appealing food images in brain areas involved in reward processing (Malik et al., 2008), and changes in ghrelin plasma levels positively correlate with peripheral 2-AG levels during consumption of hedonic food (Monteleone et al., 2012). Ghrelin-eCB interactions are further corroborated by the effects of SR141716A in fasted rats, which was shown to inhibit not only food intake but also increase in ghrelin plasma levels associated under control conditions to food deprivation (Cani et al., 2004). Moreover, a parallel and early increase of eCBs (AEA and 2-AG) and ghrelin plasma levels was shown in obese subjects upon exposure to hedonic food (Rigamonti et al., 2015). Interestingly, inhibition of 2-AG synthesis or lack of CB_1_ receptors abolishes both AMP-activated protein kinase (AMPK)-signaling and ghrelin-mediated orexigenic effects (Kola et al., 2008). Supporting the idea of a functional interaction between ghrelin and eCBs through the activity of AMPK, an intact CB_1_ signaling is required for ghrelin action (Kola et al., 2008), and likewise, eCB facilitatory effects on AMPK activity are lost in case of deletion of GHSR1a (Lim et al., 2013). Several studies reviewed in section The ghrelin-brain axis and endocannabinoid signaling: reshaping the hedonic food network are schematized in Tables 2, 3.

**Table 3:** Selected studies showing the crosstalk between DA, insulin, leptin and eCB signaling.

**DA, insulin, leptin and eCB signaling**	**Liability to hedonic food**	**Study**
Insulin-mediated inhibition on food-induced conditioned place preference	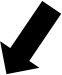	Figlewicz et al., 2004, 2006
Insulin-induced decrease of VTA DA release/intra-VTA insulin decreases palatable food intake	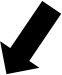	Mebel et al., 2012
Repeated exposure to palatable food impairs insulin signaling and decrease of DA clearance	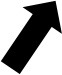	Speed et al., 2011
Intra-VTA insulin infusion enhances brain reward self-stimulation	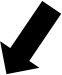	Bruijnzeel et al., 2011
Unlimited access to HFD increases 2-AG and abolish insulin-induced LTD in the VTA	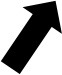	Labouèbe et al., 2013
Hyperinsulinemia disrupts insulin-induced LTD in VTA DA neurons	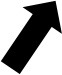	Liu et al., 2013
Insulin infusion reduces ratings of palatability but not in insulin-resistant patients	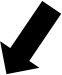	Tiedemann et al., 2017
Intranasal insulin reduces functional strength between VTA and NAC	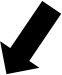	Tiedemann et al., 2017
Intra-LH leptin infusion occludes conditioned place preference associated with palatable food	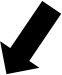	Liu et al., 2017
Excessive HFD intake blunts leptin's effects on OX neurons	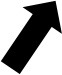	Liu et al., 2017
Stimulation of LH neurons “reveal” OX-CB_1_ receptor crosstalk and reward-induced preference	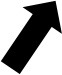	Taslimi et al., 2011; Yazdi et al., 2015
Leptin reduces sucrose rewarding and incentive value and sucrose-elicited DA signaling	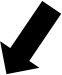	Domingos et al., 2011
Potentiation of OX1 receptor activity is abolished by CB_1_ receptor blockade or DAGL inhibition	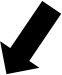	Jäntti et al., 2013

*Main effects on liability to hedonic food consumption are schematized. Ascending and descending arrows denote increase and decrease, respectively*.

## Back to dopamine: the endocannabinoid tone as gatekeeper of reward processing and the role of insulin signaling

As noted before, the potentiation of eCB tone can induce a tonic or a phasic increase of DA activity in the mesolimbic circuitry, and primarily in the NAc (Szabo et al., 2002; Cheer, 2004; Gardner, 2005; Solinas et al., 2006), because of enhanced activity of VTA DA neurons (Wu and French, 2000). The eCB-mediated fine tuning of midbrain DA-ergic activity is based on the ability to retrogradely inhibit, via activation of presynaptic CB_1_ receptors, excitatory glutamatergic terminals or inhibitory GABA-ergic terminals that synapse on VTA DA neurons (Riegel and Lupica, 2004). Thus, eCB-mediated increase of excitability of VTA neurons and midbrain signaling depends on indirect disinhibition of DA neurons via decrease of both GABA-ergic inhibition and glutamatergic excitation of DA neural activity (Szabo et al., 2002; Melis et al., 2004). Downstream to VTA DA projecting neurons, activation of presynaptic CB_1_ receptors located on glutamatergic terminals that make synapses with NAc GABA-ergic medium spiny neurons, induces inhibition of excitatory inputs to NAc and subsequent increase of inhibitory inputs to DA-ergic neurons of the VTA (Robbe et al., 2001). This is a mechanism of short-term mediated plasticity (i.e., DSI) induced by the increase of eCB tone, disinhibition of VTA DA neurons and increase of DA release within the NAc. However, in parallel, evidence for CB_1_ receptor long-term activity-dependent synaptic plasticity (i.e., LTD of glutamatergic inputs) has been reported in the NAc (Robbe et al., 2002) (Figure 1). Alterations in activity-dependent forms of synaptic plasticity such as LTD in the NAc are recognized factors in development of addictive behaviors, as well as of loss of control and impulsive behavior in animals that underwent repeated exposure to drug of abuse (Kasanetz et al., 2010). Yet, to date there is no conclusive evidence for a similar process in hedonic food-associated compulsive behavior.

So far, our review of “unusual suspect” such as leptin, ghrelin and orexin, and of their liability for hedonic eating did not include on purpose the master metabolic hormone insulin, whose function extends much far beyond glucose cellular uptake or suppression of hepatic gluconeogenesis. Indeed, once permeated the blood-brain barrier and activated its IR receptors, insulin can signal satiety to the brain (Figlewicz and Benoit, 2009) and influence the activity of VTA DA neurons (Figlewicz et al., 2003). With respect to the action of insulin on reward-driven intake of palatable food, emblematic are the studies demonstrating insulin's inhibitory effects on food-conditioned place preference (Figlewicz et al., 2004) and motivation to sucrose consumption (Figlewicz et al., 2006). Remarkably, DA homeostasis is directly affected by insulin potentiating action on DAT expression and activity; therefore, depletion of insulin reduces DAT cell-surface expression and the DA-releasing effects of amphetamine (Williams et al., 2007). Repeated exposure to HFD has been mechanistically linked to impairment of insulin signaling (i.e., insulin-activated signaling kinase), decrease of DAT cell expression and activity, DA clearance and amphetamine-mediated effects (Speed et al., 2011). Thus, insulin-induced decrease of DA release in the VTA appears attributable to an increase of DAT-mediated DA reuptake, and intra-VTA insulin has been shown to reduce intake of palatable food in sated animals (Mebel et al., 2012). Considering further that insulin delivery into the VTA enhances the threshold required to attain brain rewarding effects via intracranial self-stimulation (Bruijnzeel et al., 2011), it becomes of primary importance to understand how insulin mediates synaptic transmission onto VTA DA neurons. For this reason, it is highly relevant that insulin has been found able to induce LTD of glutamatergic (i.e., AMPA) synaptic transmission in VTA DA neurons (Labouèbe et al., 2013). Moreover, insulin-induced LTD of AMPA receptor-mediated excitatory transmission in DA-ergic neurons was shown to depend on eCB synthesis, and specifically on 2-AG synthesis, and therefore on retrograde eCB signaling and presynaptic CB_1_-mediated inhibition of glutamate release. However, insulin-induced LTD in the VTA is a very sensitive mechanism, so that unlimited access to palatable high-fat food and increase in plasma insulin temporarily abrogated insulin-associated LTD in VTA DA neurons. Such an effect depended on the increase of eCB tone that was relieved by the blockade of CB_1_ receptors (Labouèbe et al., 2013). Reducing excitatory transmission in the VTA means that insulin action is translated into a reduction of DA bursting activity and signaling in the mesocorticolimbic circuit, thus occluding reward processing and incentive salience of food-associated cues. If experimental hyperinsulinemia disrupts insulin-induced LTD in VTA DA neurons, and insulin administration fails to induce depression of DA neurons (Labouèbe et al., 2013; Liu et al., 2013), then the restraining effects of insulin on palatable food preference could not be exerted anymore (Figure 1).

Even the short-term exposure to a sweetened HFD has been shown to enhance food-seeking behavior, which was suppressed by intra-VTA insulin infusion that inhibited the increase in glutamate release and excitatory synaptic transmission onto VTA DA neurons (Liu et al., 2016). Intranasal insulin delivery in healthy subjects was shown to reduce value assignment and ratings of palatability to food pictures, while food preference scores were not reduced but showed an increasing trend in insulin-resistant individuals (Tiedemann et al., 2017). The dynamic analysis of connectivity and amplitude of blood oxygenation level-dependent (i.e., BOLD) within the mesolimbic reward circuitry showed that intranasal insulin in normoinsulinic subjects induced a reduction of the functional strength of projections between VTA and NAc. Conversely, such inhibitory action upon the functional connectivity between VTA and NAc was not observed in insulin-resistant subjects, as predicted on the basis of the insulin-induced synaptic depression of VTA DA neurons (Labouèbe et al., 2013; Liu et al., 2013; Tiedemann et al., 2017). These data support the idea that insulin signaling inhibits the DA-ergic drive from VTA to NAC, thus contributing to convey information of devaluation of palatable food and suppression of salience attribution to reward-associated food cues (Figure 1). Most of the investigations reviewed in section Back to Dopamine: The Endocannabinoid Tone as Gatekeeper of Reward Processing and the Role of Insulin signaling are schematized in Table 3.

## Irresistible food

How can hedonic food become irresistible? Probably, the simplest aspect to consider is that exposure to highly rewarding palatable food is incessant and its availability in Western societies practically unlimited. Moreover, high-rewarding palatable food is often a fat-rich food. Generally, such unrestricted access to high-fat food is considered the main characteristic of the “obesogenic environment” in Western societies. However, high-fat and sweetened food is obesogenic not only because of its caloric content but also because its consumption becomes systematic and its rewarding value takes over the nutritional need.

### Leptin

As discussed above, besides the excessive intake of high calorie food also food restriction can increase motivation for food reward. Leptin signaling appears to be involved in both aspects. Activation of leptin receptor-expressing inhibitory neurons in the LH decreases feeding, and produces an increase of VTA tyrosine hydroxylase and DA content in the NAc (Leinninger et al., 2009). On the other hand, when leptin activates leptin receptor-expressing neurons within the VTA, activity of DA neurons is reduced and food intake decreased (Hommel et al., 2006). This focuses our attention on the LH to VTA neural pathway as a key circuit involved in both energy homeostasis and brain reward. We mentioned that activation of OX neurons within the LH has been linked to environmental cues-associated drug and food reward, and reward seeking behavior (Harris et al., 2005). Recently, it has been shown that leptin may regulate body energy state via suppression of excitatory synaptic drive upon OX and MCH neurons projecting from the LH to VTA neural pathway (Liu et al., 2017). In the same study, intra-LH leptin infusion occluded HFD-associated place preference. Interestingly, both excessive energy intake (i.e., HFD) and energy depletion (i.e., fasting) blunted the effects of leptin upon OX and MCH neurons. Hence, leptin function along the LH to VTA pathway can be reduced or impaired either by a status of “leptin resistance” or by a status of hypoleptinemia. In line with this, the rewarding value of sucrose is enhanced by food restriction and leptin administration reduces its incentive value as well as sucrose-elicited DA signaling (Domingos et al., 2011). Moreover, an increased activation of brain regions involved in reward processing in response to high-calorie food pictures has been described in obese subjects, whose enhanced responsiveness to palatable food items correlates with hyperleptinemia and possible leptin resistance (Jastreboff et al., 2014) (Figure 1).

Disentangling causes from effects (Myers et al., 2010) leptin resistance is not necessarily the result of obesity, and some kinds of dietary sugars (e.g., fructose) have been shown to induce leptin insensitivity regardless of body weight gain (Shapiro et al., 2008). Although a direct proof of extra-hypothalamic (i.e., arcuate nucleus) leptin resistance is lacking, there is evidence that leptin receptor-expressing neurons in the LH regulate the VTA and the mesolimbic DA system, and that leptin-deficient subjects can meet the criteria for “food addiction” diagnosis (Albayrak et al., 2012). Thus, dietary factors even before weight gain and adiposity are key elements accountable for vulnerability of leptin-sensitive neurons. Even short-term overfeeding of high palatable diet (providing 33 and 45% of calories by fat and carbohydrates, respectively) has been shown to produce leptin and insulin resistance (Wang J. et al., 2001). Indeed, insulin elevation affects leptin signaling and hyperinsulinemia can be associated to the development of leptin resistance (Kellerer et al., 2001). Either in the case of hypoleptinemia or in that of leptin insensitivity, blunted leptin signaling in the mesolimbic DA circuitry can reduce the salience of food reward, stimulating the overconsumption of palatable food to compensate the weakening of subjective reward. Homeostatic regulation of feeding and energy balance, as well as hedonic eating are finely regulated by leptin signaling along the LH to VTA axis. Viral-mediated knockdown of leptin receptors within the LH increases caloric intake of palatable food and knockdown of leptin receptors in the midbrain increases efforts to obtain sucrose and DA transmission in the NAc (Davis et al., 2011b). These data demonstrate that the functional link between overconsumption of calorie-dense food and motivation to reward is controlled by leptin signaling along the LH to VTA neural pathway.

The existence of lipid sensing neurons involved in the constant surveillance of the nutritional state of the body allows to integrate multiple endocrine signals such as leptin and insulin to maintain energy homeostasis (Moullé et al., 2014). Intracerebral infusion of the long-chain fatty acid OA elicits satiety and decreases hepatic glucose production (Obici et al., 2002), and in addition brain inhibition of CPT1-dependent fat oxidation suppresses food intake (Obici et al., 2003; Coccurello et al., 2010). Notably, 3 days of exposure to high fat palatable diet or high sucrose diet was reported to blunt these anorectic effects induced by OA (Morgan et al., 2004). Accordingly, not only macronutrient composition (both lipids and carbohydrates) but also caloric load is accountable for the rapidly acquired insensitivity to OA-induced satiation. OA-responsive neurons have been reported for the first time in LH (Oomura et al., 1975), and FA and glucose sensitive neurons regulating food intake and insulin response are well-described within the ventromedial and arcuate nuclei of the hypothalamus (Moullé et al., 2014). Now, there is evidence for lipid sensitive neurons within the VTA and for DA neurons that can detect and transport FAs intracellularly; moreover, intra-VTA infusion of OA decreases DA signaling and reward-seeking behavior (Hryhorczuk et al., 2017). It would be then of primary interest to ascertain whether short-term exposure to palatable high fat or high sucrose diet may blunt the effects of monounsaturated FAs and disinhibit DA signaling and reward-seeking behavior. Similarly, it would be interesting to determine whether intra-VTA infusion of saturated FAs such as palmitic acid may increase mesolimbic DA signaling and derange the inhibitory effects exerted by leptin and insulin signaling.

### Ghrelin, eCBs, and orexin cross-talk

By contrast, inverse functional effects are described when ghrelin is administered centrally, or directly infused into the VTA. Intracerebroventricular ghrelin enhances food reward-driven instrumental behavior (Skibicka et al., 2012), and these effects of ghrelin on reward-associated salient stimuli are consistent with the effects of ghrelin on potentiation of VTA DA neural activity (Abizaid et al., 2006), and the increase of DA release in the NAc after intra-VTA ghrelin infusion (Jerlhag et al., 2007). Additionally, the increase of sucrose-driven instrumental behavior after intra-VTA ghrelin infusion provides convincing evidence for a role of ghrelin signaling in reward-motivated feeding via VTA DA neurons (Skibicka et al., 2011). Remarkably, increased effort and motivation to obtain palatable food can be further boosted by intra-VTA chronic ghrelin infusion both in food-restricted and in satiated animals (King et al., 2011). In an independent study, intra-VTA ghrelin was shown to enhance both hyperphagia induced by fasting and reward-associated feeding in sated animals (Wei et al., 2015). Again, as noted for leptin signaling, homeostatic and hedonic feeding appear integrated one another by the action of ghrelin at VTA DA neurons. In support of the idea that ghrelin elicits food addiction-like behavior is the demonstration that changes in ghrelin levels have commonalities with addiction to substance abuse. Thus, after extinction of instrumental behavior to obtain food reward, intra-VTA ghrelin chronic infusion was shown to reinstate cue-induced responses for palatable chocolate pellets (St-Onge et al., 2016). The fact that ghrelin signaling within the VTA can facilitate relapse to food reward is a compelling demonstration that drug abuse and hedonic eating share indeed several mechanisms of action. An interesting interpretation of ghrelin role in reward is that ghrelin signaling can alter the “set point” of VTA DA neurons, thus potentiating the activation of midbrain DA induced by natural or artificial rewards, as well as by cue-associated rewards (Dickson et al., 2011).

As noted above, the increase of hypothalamic AMPK activity appears an obligatory step in the orexigenic effects mediated by ghrelin and eCBs (Kola et al., 2005), and ghrelin effects are abolished in case of disruption of eCB signaling (Kola et al., 2008). In turn, the effects of eCB on AMPK activation could not be exerted in the absence of an intact ghrelin signaling (Lim et al., 2013). eCB signaling is therefore at the crossroad between AMPK brain activity and ghrelin-mediated orexigenic and rewarding effects, and changes in brain eCB levels may have a deep impact on food addiction-like behavior.

Considering that eCBs derive from FAs, and that dietary composition of FAs can significantly affect the synthesis and brain levels of eCBs (Artmann et al., 2008), the conundrum of hedonic eating appears tightly intertwined with that of overeating, overweight, dysregulation of endocrine factors and, ultimately, obesity. Overactivity of the ECS in obesity and insulin resistant subjects is well-documented (Maccarrone et al., 2010b; Battista et al., 2012) and, interestingly, there are reports showing that AEA levels do not decrease after meal in obese individuals as they do in normal weight subjects (Gatta-Cherifi et al., 2012). Eating or not palatable hedonic food makes a great difference in terms of derangement of the ECS. Indeed, consumption of palatable food increases 2-AG plasma levels that, conversely, decrease in obese subjects consuming non-palatable food (Monteleone et al., 2016). Moreover, not only in obese patients but also in normoweight subjects 2-AG plasma levels have been reported to increase even before (as well as after) exposure to palatable food (Monteleone et al., 2012), providing an interesting evidence for a role of 2-AG in reward anticipation and processing. Reward-oriented preference for palatable food was found associated with the increase of hypothalamic 2-AG after only 3 days of exposure to HFD (Higuchi et al., 2011). However, the latter condition induced a transient increase of 2-AG, 2 weeks of exposure to high fat food elicited a persistent increase of hypothalamic 2-AG that in turn elicited food-associated conditioned food preference (Higuchi et al., 2012). In the same study, prolonged exposure to high fat food induced an increase of hypothalamic glial fibrillary acid protein (GFAP) expression levels that was reduced, together with food preference, by CB_1_ receptor blockade. Overall, it was hypothesized that 2-AG may function as inflammatory stimulus triggering persistent astrocyte activation and development of preference for salient stimuli, associated with palatable food (Higuchi et al., 2012).

The increase of hypothalamic eCB signaling induced by HFD eating allows to reconnect the homeostatic regulation of food intake to reward-dependent overeating and reinforcing properties of hedonic food. We might portray the LH as a neural substrate interfacing homeostatic information, such as that carried by leptin and ghrelin, with the neural network involved in reward processing (i.e., mesolimbic circuit). Hence, reward processing and homeostatic regulation of feeding are functions both attributed to LH. As discussed earlier, the high integrative function of LH is well illustrated by the circuit between LH GABA-ergic neurons that project to VTA GABA neurons and, in turn, synapse and disinhibit the activity of VTA DA cells. The stimulation of LH GABA-ergic neurons elicits a strong eating response (Jennings et al., 2015), as well as a reward-associated feeding behavior (Nieh et al., 2016). Several studies reported the functional crosstalk between OX and CB_1_ receptors within the VTA that are revealed upon LH stimulation, and the facilitatory effects on reward-oriented preference (Taslimi et al., 2011; Yazdi et al., 2015). Different incoming excitatory inputs converge to LH from cortical and subcortical structures like, for instance, medial prefrontal cortex and hippocampus (Cenquizca and Swanson, 2006; Reppucci and Petrovich, 2016). Similarly, several inhibitory GABA-ergic afferents reach LH from amygdala and NAc (Hahn and Swanson, 2012; Reppucci and Petrovich, 2016). CB_1_ receptors are densely distributed along the entire hypothalamus, and both symmetrical and asymmetrical synapses are established with LH neurons via axon terminals rich in CB_1_ receptors (Tsou et al., 1998; Wittmann et al., 2007). The increase of eCB signaling can consequently exert a powerful retrograde inhibition on excitatory and inhibitory afferent terminals thus dictating and shaping information processing within the LH circuits. For instance, the increase of OX signaling potentiates the excitatory transmission in VTA DA neurons that influence motivation (e.g., seeking behavior) and reward-induced reinforcement (Borgland et al., 2009). In turn, the increase of activity of VTA DA neurons stimulate D1 receptors located on NAc GABA-ergic neurons and increase inhibition on LH GABA-ergic cells, to an extent that ultimately depends on the activation of presynaptic CB_1_ receptors. Remarkably, recent data favor the concept of a tight functional interplay between OX signaling and eCB release. Stimulation of OX-A has been shown to induce an overflow of ARA through cytosolic phospholipase A2 (PLA2) and diacylglycerol lipase (DAGL) cascades (Turunen et al., 2012), which are responsible for ARA release and 2-AG synthesis, respectively (Gijón and Leslie, 1999; Bisogno et al., 2003). Moreover, the effects induced by the stimulation of OX receptor 1 (OX1) were enhanced after co-expression of OX1 and CB_1_ receptors in the same cells (Jäntti et al., 2013). Consistently, potentiation of OX1 activity was abolished by CB_1_ receptor blockade or DAGL inhibition, thus suggesting that OX1-mediated activity was boosted via 2-AG release and autocrine CB_1_ signaling (Jäntti et al., 2013). Collectively, these findings provide a novel and interesting mechanistic explanation for the functional interaction between OX and ECS systems, via a communication path that engages OX1 receptor activation, DAGL release and 2-AG synthesis, terminating with the autocrine stimulation of CB_1_ receptors (Figure 1).

### Lateral hypothalamus, orexin-, and dopamine-ECS communication

Altogether, these mechanisms add an important piece of evidence for the causal role of eCB signaling in food reward processing within the LH local microcircuits, as well as in the extended neural network encompassing cortical and subcortical structures and mesolimbic DA system. A large body of evidence corroborates the idea that OX neurons in the LH integrate endocrine signals such as leptin and ghrelin (Yamanaka et al., 2003; Liu et al., 2017) with reward-seeking behavior, including food reinforcement and hedonic eating (Harris et al., 2005; Borgland et al., 2009; Cason et al., 2010; Sharf et al., 2010).

Development of context-conditioned expectation for hedonic food is required for the expression of food addiction-like behavior, and OX neurons are activated by external cues anticipating the availability of palatable food (Harris et al., 2005; Choi et al., 2010). Consumption of the latter by sated animals is a sign of compulsive eating, and blockade of OX1 receptors was shown to reduce reward-associated HFD consumption in sated rats (Choi et al., 2010). We have formerly discussed the role of ghrelin signaling mainly because its orexigenic and reinforcing effects are mediated via the activation of OX neurons, and because ghrelin links OX neurons to the midbrain activity of VTA neurons and DA release (Abizaid et al., 2006; Borgland et al., 2009; Jerlhag et al., 2010). Consequently, ghrelin is not only an appetite-stimulating factor and a homeostatic signal transmitting information of negative energy balance. Indeed, assign a rewarding value to food involves ghrelin signaling and the facilitatory effects of ghrelin on reward-driven food consumption is a process that requires the integrity of OX signaling (Perello et al., 2010).

Today, there is a recognized link between psychosocial stress, drive to eat, consumption of palatable food and fat accretion that should provide relief from discomfort (Dallman et al., 2005; Coccurello et al., 2009), although chronic stress and social subordination may have opposite effects on energy balance and macronutrient selection (Moles et al., 2006; Coccurello et al., 2017). Stress increases not only the impact of incentive salience of drug of abuse (Koob and Volkow, 2017), but also the rewarding value of palatable food (Adam and Epel, 2007). In the context of food addiction-like behavior, it seems crucial to understand the mechanisms underlying relapse to excessive intake of high fat palatable food, in spite of its negative consequences. Akin to drug addiction, relapse or reinstatement of previous food-seeking behavior is the main threat to the possibility to fight eating addiction. Exposure to different stressors can reinstate high motivation for food craving and unhealthy food-seeking behavior even in lack of food availability (Calu et al., 2014). An important signaling cascade involving OX1 receptors, generation of 2-AG and disinhibition of VTA DA neurons has been recently disclosed in stress-induced reinstatement of previously extinguished preference for cocaine (Tung et al., 2016). This study elegantly shows that stress-induced activation of LH OX neurons leads to OX release and activation of postsynaptic OX1 receptors on the VTA DA-ergic neurons, and that OX1 receptor activation triggers the production of 2-AG and the subsequent retrograde inhibition of GABA release via presynaptic CB_1_ receptors. By means of this mechanism, the cascade LH-OX/OX1/2-AG/CB_1_ inhibits presynaptic GABA release and activates VTA DA neurons, leading to stress-induced relapse to cocaine. The latter mechanism appears consistent with the most solid data discussed herein with respect to the role of ghrelin-OX-eCB-DA signaling in the LH-VTA-NAc neural circuit, and its pathophysiological meaning for food addiction-like behavior (Figure 1). Some of the investigations reviewed in the present section are schematized in Tables 2, 3.

## Concluding remarks

Food-seeking activity, reward-oriented behavior, food hunting, motivation to obtain palatable food and compulsive eating are all determinants of food-addiction like behavior, especially when hedonic calorie-dense food is ubiquitous and there is externally imposed or voluntary food restriction, as in the case of obesity or eating disorders. Future efforts should be focusing more on the interplay between leptin, insulin and ghrelin signaling along the lateral hypothalamus-mesolimbic DA axis. The study of the alterations induced by consumption of hedonic food of different dietary composition, conditioning to food-associated external cues, and impaired inhibitory control over food craving are all aspects in which changes of the eCB tone may have an important heuristic significance. A major understanding of the mechanisms by which variations in eCB signaling can control the “weight” of incoming and outcoming inputs in the LH-VTA axis, thus modifying its synaptic architecture, will provide novel insights in the fight against hedonic food-associated diseases.

## Author contributions

RC and MM: conceived the study; RC: wrote the manuscript, that was revised by MM.

### Conflict of interest statement

The authors declare that the research was conducted in the absence of any commercial or financial relationships that could be construed as a potential conflict of interest.

## Abbreviations

2-AG: 2-arachidonoylethanolamine
AEA: *N*-arachidonylethanolamine (anandamide)
AMPK: AMP-activated protein kinase
AN: anorexia nervosa
AgRP: agouti-related peptide
ARA: arachidonic acid
ARC: arcuate nucleus
BOLD: blood oxygenation level-dependent
CB_1_: cannabinoid receptor 1
CPT1: carnitine palmitoyltransferase-1
D2Rs: D2 receptors
DA: dopamine
DAGL: diacylglycerol lipase
DHA: docosahexaenoic acid
DHEA: docosahexaenoylethanolamide
DSE: depolarization-induced suppression of excitatory
DSI: depolarization-induced suppression of inhibitory
eCBs: endocannabinoids
ECS: endocannabinoid system
EPA: eicosapentaenoic acid
EPEA: *N*-eicosapentaenoylethanolamine
GHSR: growth hormone secretagogue receptor
HFD: high fat diet
IRs: insulin receptors
LEA: *N*-linoleoylethanolamine
LepRb: leptin receptors
LH: lateral hypothalamus
LNA: linoleic acid
LTD: long-term depression
MCH: melanin-concentrating hormone
MUFAs: monounsaturated fatty acids
NAc: nucleus accumbens
NAPE: *N*-acylphosphatidylethanolamine
NPY: neuropeptide Y
OEA: *N*-oleoylethanolamine
OX: orexin
PEA: *N*-palmitoylethanolamine
PLA2: phospholipase A2
POMC: proopiomelanocortin
PFC: prefrontal cortex
PUFAs: polyunsaturated fatty acids
SEA: *N*-stearoylethanolamine
SFAs: saturated fatty acids
STD: short-term depression
VTA: ventral tegmental area.
